# The Effect of Calcium on the Cohesive Strength and Flexural Properties of Low-Methoxyl Pectin Biopolymers

**DOI:** 10.3390/molecules25010075

**Published:** 2019-12-24

**Authors:** Christine Byun, Yifan Zheng, Aidan Pierce, Willi L. Wagner, Henrik V. Scheller, Debra Mohnen, Maximilian Ackermann, Steven J. Mentzer

**Affiliations:** 1 Laboratory of Adaptive and Regenerative Biology, Brigham & Women’s Hospital, Harvard Medical School, Boston, MA 02115, USA; cbyun2018@health.fau.edu (C.B.); yifan.a.zheng@gmail.com (Y.Z.); afpierce@bwh.harvard.edu (A.P.); willi.wagner@uni-heidelberg.de (W.L.W.); 2 Department of Diagnostic and Interventional Radiology, Translational Lung Research Center, University of Heidelberg, 69115 Heidelberg, Germany; 3 Joint BioEnergy Institute, Emeryville CA and the Environmental Genomics and Systems Biology Division, Lawrence Berkeley National Laboratory, Berkeley, CA 94701, USA; scheller@berkeley.edu; 4 Complex Carbohydrate Research Center and Department of Biochemistry and Molecular Biology, University of Georgia, Athens, GA 30602, USA; dmohnen@ccrc.uga.edu; 5 Institute of Functional and Clinical Anatomy, University Medical Center of the Johannes Gutenberg-University Mainz, 55131 Mainz, Germany; maximilian.ackermann@uni-mainz.de

**Keywords:** polysaccharides, material properties, hydration, methoxylation, fracture mechanics

## Abstract

Pectin binds the mesothelial glycocalyx of visceral organs, suggesting its potential role as a mesothelial sealant. To assess the mechanical properties of pectin films, we compared pectin films with a less than 50% degree of methyl esterification (low-methoxyl pectin, LMP) to films with greater than 50% methyl esterification (high-methoxyl pectin, HMP). LMP and HMP polymers were prepared by step-wise dissolution and high-shear mixing. Both LMP and HMP films demonstrated a comparable clear appearance. Fracture mechanics demonstrated that the LMP films had a lower burst strength than HMP films at a variety of calcium concentrations and hydration states. The water content also influenced the extensibility of the LMP films with increased extensibility (probe distance) with an increasing water content. Similar to the burst strength, the extensibility of the LMP films was less than that of HMP films. Flexural properties, demonstrated with the 3-point bend test, showed that the force required to displace the LMP films increased with an increased calcium concentration (*p* < 0.01). Toughness, here reflecting deformability (ductility), was variable, but increased with an increased calcium concentration. Similarly, titrations of calcium concentrations demonstrated LMP films with a decreased cohesive strength and increased stiffness. We conclude that LMP films, particularly with the addition of calcium up to 10 mM concentrations, demonstrate lower strength and toughness than comparable HMP films. These physical properties suggest that HMP has superior physical properties to LMP for selected biomedical applications.

## 1. Introduction

A variety of polysaccharide polymers have been implicated in biomedical applications, including alginate [1], cellulose [2], chitin [3], agarose [4], and pectin [5]. Pectin is a particularly interesting polysaccharide because of its structural and functional features. Chemically, commercial pectins contain primarily linear chains of homogalacturonan, a partially methyl esterified polymer of (1-4)-α-D-galacturonic acid (GalA) [6], along with lesser amounts of rhamnogalacturonan [7]. Pectin in plants is more complex, with regions of homogalacturonan covalently linked to the branched pectic polymers rhamnogalacturonan I and rhamnogalacturonan II [8] and to arabinogalactan proteins [9]. Pectin demonstrates remarkable adhesivity to gut mucins, providing a useful mechanism for oral drug delivery [10]. Pectin also binds the mesothelial glycocalyx of visceral organs [5], suggesting its potential role as a mesothelial sealant [11].

A variety of pectin polymers can be produced, depending on the plant source from which the pectin is extracted. Commonly, citrus peels and apple pomace are the raw materials used for pectin manufacturing. After pretreating them to remove contaminating sugars, the citrus peels are extracted in acidified water. The low pH dissociates ionic linkages and hydrolyzes glycosidic bonds. The extraction conditions also hydrolyze ester linkages, leading to variable reduction in the degree of methyl esterification. The result is pectin extraction products with variable degrees of methoxylation. The degree of methoxylation is generally distinguished as below 50% (low-methoxyl pectin, LMP) or above 50% (high-methoxyl pectin, HMP). Although the absolute distinction between LMP and HMP pectins is somewhat arbitrary [12], the degree of methoxylation is associated with physical properties and sensitivity to calcium concentrations [13,14]. 

Previous functional studies of LMP have focused on specialized pectin preparations used in food and pharmaceutical industries. In the food industry, the gelling and thickening properties of LMP are used in jams and jellies [12]. In these applications, gelation can be facilitated by lowering the temperature [15], adding sucrose [16] and, in the context of LMP, adding calcium [17]. LMP and calcium have provided a valuable low calorie dietetic substitute for conventional food products [18].

For the pharmaceutical industry, an intriguing feature of the LMP is its “egg-box” structure. Based on the optical activity of the pectin polymers, Morris et al. proposed a corrugated structure composed of two or more buckled chains linked by ion-binding [19,20]. The egg-box structure of pectin has been advocated as a potential drug delivery system [21]. Functionally, pectin demonstrates remarkable adhesivity to gut mucins, providing a useful mechanism for controlled oral drug delivery [10]. Pectin also binds the mesothelial glycocalyx of visceral organs [5], suggesting its potential role as a mesothelial sealant [11].

In both the food and pharmaceutical industries, the focus of these studies has been on the process of gelation; surprisingly little is known about the physical properties of cured pectin films. To investigate the functional properties of LMP films relevant to biomedical applications, we studied the physical and mechanical properties of LMP films at a variety of calcium concentrations and hydration states.

## 2. Results

### 2.1. LMP and HMP Polymer Films

**Figure 1:**
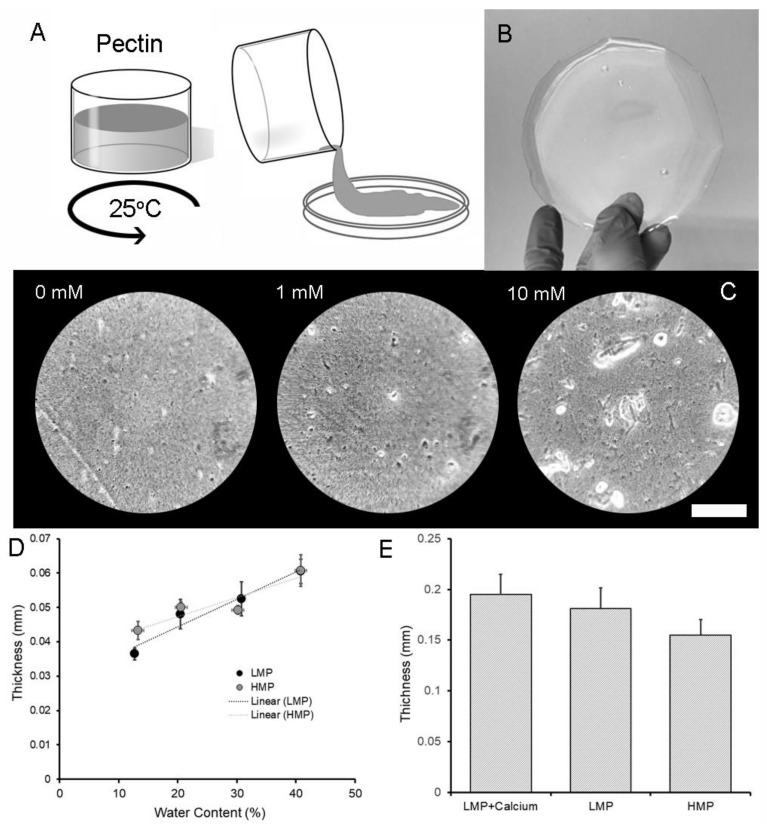
Preparation of pectin polymers. (**A**) Powered pectin with low methyl esterification (less than 50%) was prepared in a high-shear mixer and poured into a mold for curing and subsequent analysis. (**B**) After curing to a 10% water content, the low-methoxyl pectin (LMP) films were grossly transparent. (**C**) With the addition of calcium, microscopic examination demonstrated an increased number of microaggregates with an increasing calcium concentration (Bar = 250 µm). When compared to pectin films with high methyl esterification, the thickness of the LMP was similar to high-methoxyl pectin (HMP) at a range of water contents (LMP R^2^ = 0.855; HMP R^2^ = 0.949) (**D**). The addition of 10 mM calcium to the LMP resulted in a slightly thicker film than the HMP film at a water content of 30 ± 2% (*w*/*w*) (*p* < 0.05) (**E**). Error bars = 1 SD.

HMP and LMP polymer preparation was similar. The films were dissolved in water and dispersed using high-shear mixing (Figure 1A) and cured in a controlled humidity environment. The resulting LMP films demonstrated a translucent appearance similar to cured HMP films (Figure 1B). Microscopy of the LMP films demonstrated some irregularities, particularly with the addition of calcium (Figure 1C). The LMP and HMP films demonstrated a comparable thickness over a range of hydration states (Figure 1D). Unique to LMP films, however, the addition of calcium during preparation resulted in LMP films that were significantly thicker than HMP films (*p* < 0.05) (Figure 1E). 

### 2.2. Strength and Extensibility of LMP Films

**Figure 2:**
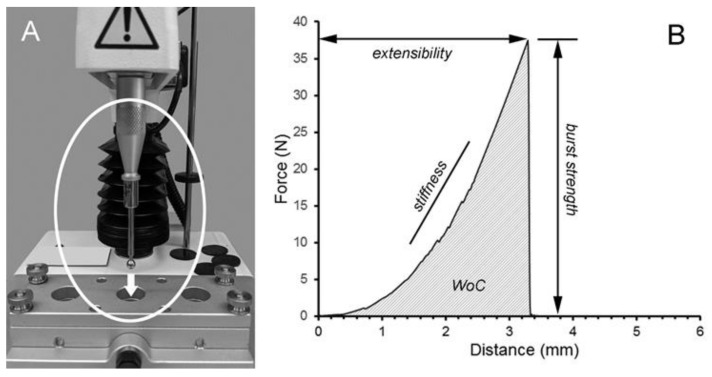
Burst strength of low-methoxyl pectin (LMP) films. (**A**) To test the cohesive properties of the LMP films, a uniaxial constant velocity was applied normal to the plane of the film until rupture. In these experiments, a 5 mm stainless steel probe was used to assess the burst strength, extensibility, stiffness, and work of cohesion (WoC) (**B**).

**Figure 3:**
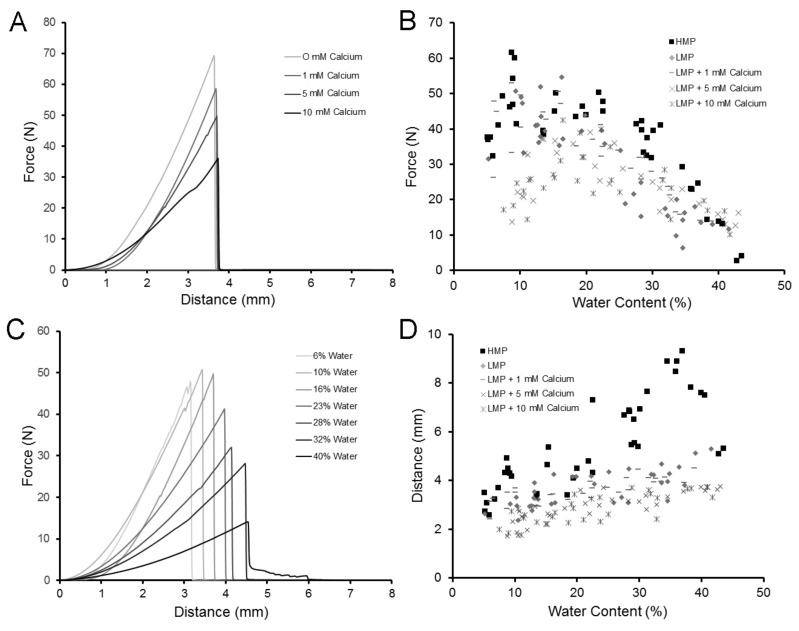
Effect of calcium concentration and water content on the burst strength and extensibility of LMP films. (**A**) In films with identical degrees of methoxylation and a similar hydration state, the addition of calcium resulted in decreased burst strength. (**B**) The burst strength of the LMP films was generally less than that of HMP, regardless of the calcium concentration or water content. (**C**) Increasing the water content of LMP films (without calcium) resulted in a decreased burst strength, but increased extensibility. (**D**) Similar to burst strength, the extensibility of the HMP films was greater than that of LMP films, regardless of the calcium concentration or water content.

The burst strength of the LMP films was measured with a uniaxial load applied at constant velocity normal to the plane of the film until rupture (Figure 2). Using these fracture mechanics, the burst strength of the LMP films decreased with an increasing calcium concentration (Figure 3A). Regardless of the calcium concentration, the burst strength of LMP films was lower than that of HMP films at a variety of hydration states (Figure 3B). Water content also influenced the extensibility of LMP films. The LMP films demonstrated an increased extensibility (probe distance) with an increasing water content (Figure 3C). Similar to the burst strength, the extensibility of the LMP films was less than that of HMP films (Figure 3D).

### 2.3. Flexural Properties of LMP Films

**Figure 4:**
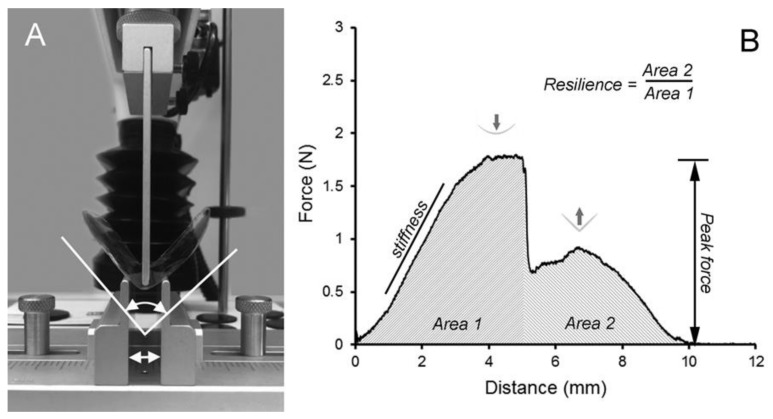
Flexural properties of LMP films. (**A**) To test the flexural properties of the LMP films, a 3-point custom bend fixture with 12 mm separation (double arrow) of the support blades was used. The upper blade was positioned equidistant from the support blades and was applied at constant velocity (2 mm/s) for a distance of 5 mm after film contact. (**B**) The applied force required for a 5 mm displacement was recorded in Area 1. The force recorded on blade withdrawal (storage modulus) was recorded in Area 2. Resilience was calculated as the ratio of Area 2/Area 1.

**Figure 5:**
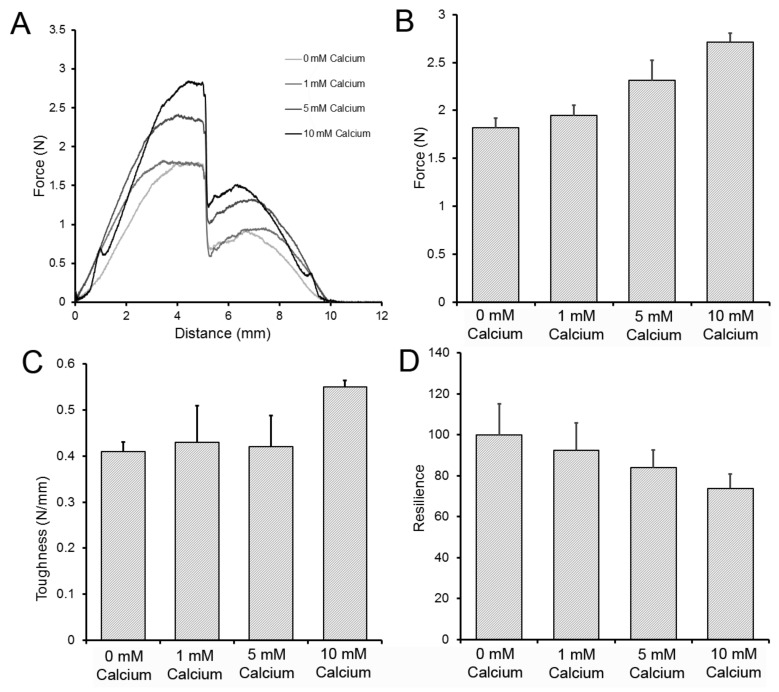
Effect of the calcium concentration on LMP film 3-point bend testing. (**A**) Representative 3-point bend tracings of LMP films with 0, 1, 5, and 10 mM calcium concentrations. (**B**) The peak force required for 5 mm blade displacement was significantly greater in LMP films with 5 and 10 mM calcium concentrations (*p* < 0.001). (**C**) Toughness, here measuring the resistance to deformation, was significantly greater in the 10 mM calcium films (*p <* 0.01). (**D**) The resilience, reflecting the ratio of the work of displacement and its corresponding stored energy, demonstrated that the resilience of 0 mM calcium films was significantly greater than that of the 10 mM calcium film (*p* < 0.01). Error bars = 1 SD.

To test the flexural properties of the LMP films, we used a 3-point bend test with dimensions reflecting common biomedical applications (e.g., a 12 mm surgical trocar) (Figure 4). The films were tested with the probe blade moving a standard 5 mm distance, with force measurements recorded during deformation and withdrawal. The 3-point bend experiments demonstrated that the force required to displace the LMP increased with an increased calcium concentration (*p* < 0.01) (Figure 5A,B). Toughness, here reflecting deformability (ductility), was variable, but increased with an increased calcium concentration. Consistent with these findings, the resilience of the films as defined in Figure 5 decreased with an increasing calcium concentration (*p* < 0.01) (Figure 5D).

### 2.4. Physical Properties of LMP Films

**Figure 6:**
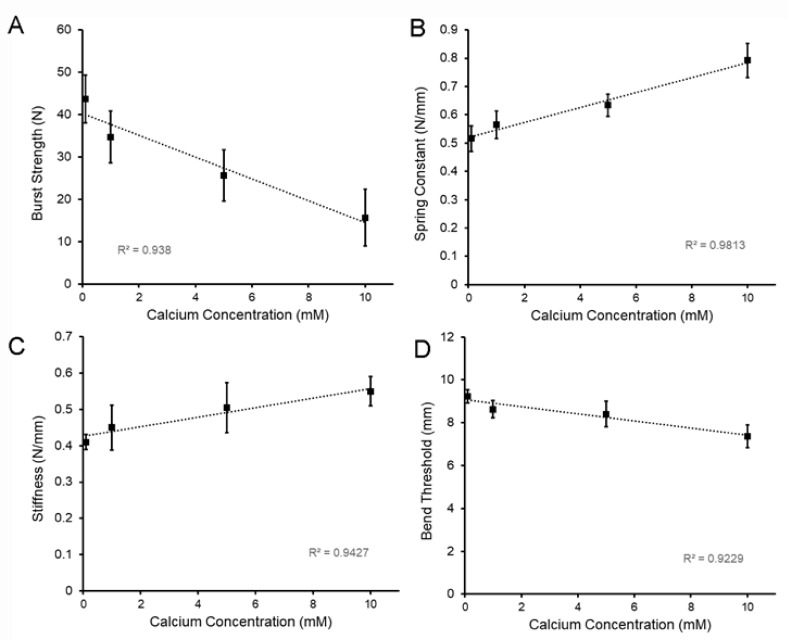
Effect of the calcium concentration on the physical properties of LMP films. The LMP films were cured to a 15.7 ± 6.6% (*w*/*w*) water content; mean values of a minimum of 10 films are shown. (**A**) Burst strength of the films, reflecting the fracture resistance to a uniaxial load applied at a velocity of 0.5 mm/s (see Figure 3), decreased with an increasing calcium concentration (R^2^ = 0.938). (**B**) The spring constant, measured as the film displacement and applied force in the 3-point bend test, increased with an increasing calcium concentration (R^2^ = 0.9813). (**C**) Similarly, the stiffness, measured as the resistance to both the fracture probe and the 3-point bend test (see Figure 2 and Figure 4), increased with an increasing calcium concentration (R^2^ = 0.9427). (**D**) The bend threshold, measured as the probe distance at the yield point in a modified 3-point bend test, decreased with an increasing calcium concentration (R^2^ = 09229). Error bars = 1 SD.

The effect of calcium on the physical properties of LMP films was studied with calcium concentrations of 1 to 10 mM. The cohesive strength of the polymers significantly decreased with an increasing calcium concentration (*p* < 0.01) (Figure 6A). The spring constant, measured as the deformation distance per unit force in the flexural testing, increased with an increasing calcium concentration (*p* < 0.01) (Figure 6B). Stiffness, derived from both the burst testing and 3-point bend test, increased with an increasing calcium concentration (Figure 6C). Finally, the bend threshold, reflecting the yield point of the film in flexural testing, decreased with an increasing calcium concentration (*p* < 0.01) (Figure 6D).

## 3. Discussion

In this report, we studied the physical properties of LMP relevant to its role as a mesothelial sealant. When compared to HMP films, we found that LMP films demonstrated lower burst strength, limited extensibility, and diminished deformability. These trends were amplified with the addition of calcium. We conclude that LMP films, particularly with the addition of calcium up to 10 mM concentrations, demonstrate lower strength and toughness than HMP films.

Pectin is conventionally characterized by the degree of methyl esterification because the mechanism of gel formation is different in high- and low-methoxyl pectins [14]. In high-methoxyl pectins, typically defined as exhitbiting greater than 50% esterification, the polymer chains interact by hydrogen bonding and hydrophobic interactions [13,22]. In low-methoxyl pectins, defined as demonstrating less than 50% esterification [14], the pectin chains interact by hydrogen bonding and fewer hydrophobic interactions. Although lower degrees of methyl esterification compromise the contributions of hydrophobicity, the tradeoff is that chain interactions can be more effectively augmented by ionic cross-linkages via calcium bridges [23,24]. Our findings demonstrated that these tradeoffs result in pectin films with a lower strength and toughness.

Potentially relevant to these physicochemical interactions was the effect of calcium on the physical properties of LMP. The addition of calcium to LMP produced a thicker gel with a decreased cohesive strength. The thicker gel may reflect the unique configuration of the proposed corrugated “egg-box” structure of LMP [20,23]. Although other factors such as chain length, molecular weight homogeneity, and methyl ester distribution are important [25,26], our studies underscore the significant effect of calcium on the functional properties of LMP.

Cohesive strength is a particularly relevant functional property of pectin-based sealants. The work of LMP cohesion, represented graphically by the area under the force-distance curve, was diminished by both the addition of calcium and an increased water content. Vinthanage et al. noted a similar finding, showing that LMP films demonstrated a significantly lower “puncture” resistance than HMP films [27]. Kyomugaso et al. described an increased microstructural density association with decreased methoxylation and an increased cation concentration [28]; however, this effect on network formation was not directly linked to gel strength [29,30].

An intriguing physical property of pectin is its elasticity. In the middle lamella of plants, pectin forms a strong but elastic matrix that can accommodate changes in turgor. In biomedical applications, the pectin biopolymer must respond to changes in organ volume. To assess the response of different pectin films to deformation, we used the 3-point bend test. In contrast to rheometric oscillatory stress, the 3-point bend procedure tests the physical properties relevant for practical application; for example, our testing fixture mirrored the geometry of surgical trocars used in clinical deployment. Consistent with observations in another system [28], the 3-point bend test demonstrated that the LMP films were stiffer than HMP films. Further, the resilience, or stored energy of the film, was further compromised with an increased calcium concentration. 

Finally, the physical properties in this study, such as strength and flexibility, are particularly relevant for mesothelial sealants [5]. The mesothelium is the surface layer of visceral organs, including the lungs, heart, bowel, and liver. These organs are notable for repetitive motion during normal activities, such as tidal ventilation, cardiac contraction, and bowel peristalsis. Even the liver is influenced by diaphragmatic descent during normal breathing. Mesothelial sealants need to not only adhere to the mesothelium, but effectively accommodate the motion and volume changes associated with normal visceral organ function. Our current results suggest that the physical properties of LMP films are less capable of adapting to these stresses than HPM films.

## 4. Methods 

### 4.1. Pectin

The citrus pectins used in this study were obtained from a commercial source (Cargill, Minneapolis, MN, USA). As previously described [31], the proportion of galacturonic acid residues in a methyl ester form determined the degree of methoxylation. In this study, the high-methoxyl pectins (HMP), defined as those pectin polymers with a greater than 50% degree of methoxylation, had a mean of 72 ± 5%. Low-methoxyl pectins (LMP), defined as polymers with a less than 50% degree of methoxylation, had a mean of 35 ± 3%. The pectin powder was stored in low humidity at 25 °C.

### 4.2. Pectin Dissolution in Water

The pectin powder was gradually dissolved at 25 °C to facilitate fluidization and dissolution, as previously described [32]. Briefly, the dissolution of pectin was achieved by a high-shear 10,000 rpm rotor-stator mixer (L5M-A, Silverson, East Longmeadow, MA USA). The dissolved pectin was poured into molds standardized for subsequent studies [33]. 

### 4.3. Humidification Chamber 

Water content of the films was maintained with a custom designed 5.7 L translucent polycarbonate humidification chamber constructed to be air-tight when sealed, as well as compatible with our material analysis instruments. Humidification within the chamber was produced by an ultrasonic humidifier or manual aerosol device. The chamber was monitored by wireless (Bluetooth) hygrometer and thermometer sensors (SensorPush, Brooklyn NY, USA). 

### 4.4. Adhesion Testing 

Polymer-polymer adhesion experiments were performed with a force-calibrated custom fixture designed for the TA-XT plus (Stable Micro Systems, Godalming, Surrey, UK). The fixture was composed of a 30 mm diameter flat-ended cylindrical probe and a flat fixture surface; both surfaces used vacuum-controlled polymer fixation. The cylindrical probe compressed the two polymers, followed by the separation of the probe from the surface by an applied tensile load. The probe velocity, compression force, and distance were recorded at 500 pps. A minimum of N = 10 films per data point were tested.

### 4.5. Film Thickness

Film thickness was determined with the TA-XT plus (Stable Micro Systems) materials analyzer, with a 5 kg load cell. After distance and load cell calibration, the spherical probe descended (0.2 mm/s) until the surface of the film was detected at a 1 gm trigger force. Film thickness was determined with a reproducible distance resolution of 1 μm.

### 4.6. Fracture Mechanics

To determine the fracture mechanics of the pectin, the biopolymers were subjected to a controlled uniaxial load normal to the plane of the polymer film using the TA-XT plus (Stable Micro Systems). A 5 mm stainless steel spherical probe was mounted on a 5 kg load cell and positioned centrally over the biopolymer. The probe compressed the biopolymers at a test speed of 1 mm/s until fracture. The fracture force and distance were recorded at 500 pps. Burst strength was defined as the peak force required for film fracture. The distance the probe traveled between polymer contact (detection at 1 N) and film fracture was defined as the extensibility (mm). The slope of the initial linear portion of the burst curve was defined as the stiffness (N/mm). 

### 4.7. Bend Properties of Pectin Films.

To test the bend properties of the LMP films, a 3-point custom bend fixture with 12 mm separation (double arrow) of the support blades was used. The upper blade was positioned equidistant from the support blades and was applied at constant velocity (2 mm/s) for a distance of 5 mm after film contact. The applied force required for a 5 mm displacement was recorded in Area 1. The force recorded on blade withdrawal (storage modulus) was recorded in Area 2. Resilience was calculated as the ratio of Area 2/Area 1. A measure comparable to extensibility is the 3-point bend threshold; that is, the distance traveled to reach the polymer yield point.

### 4.8. Statistical Analysis

The statistical analysis was based on measurements of at least three different samples. The unpaired Student’s t test for samples of unequal variances was used to calculate statistical significance. The data was expressed as the mean ± one standard deviation (SD). The significance level for the sample distribution was defined as *p* < 0.01.

## Abbreviations

HMP	high-methoxyl pectin
LMP	low-methoxyl pectin
W_c_	water content
WoC	work of cohesion
SD	standard deviation
